# Supplementary material for: Causal interplay between lactose intolerance and gut microbiota: a combined bidirectional Mendelian randomization and in vivo validation study
Source: Front Nutr. 2026 Jun 1;13:1803337. doi: 10.3389/fnut.2026.1803337 (PMC13265576; doi:10.3389/fnut.2026.1803337)
Supplement: Supplementary file 1 [file Data_Sheet_1.zip › supplementary materials/Forward/leave one out cross validation/ebi-a-GCST90027576.finngen_R12_E4_LACTONAS.pdf]

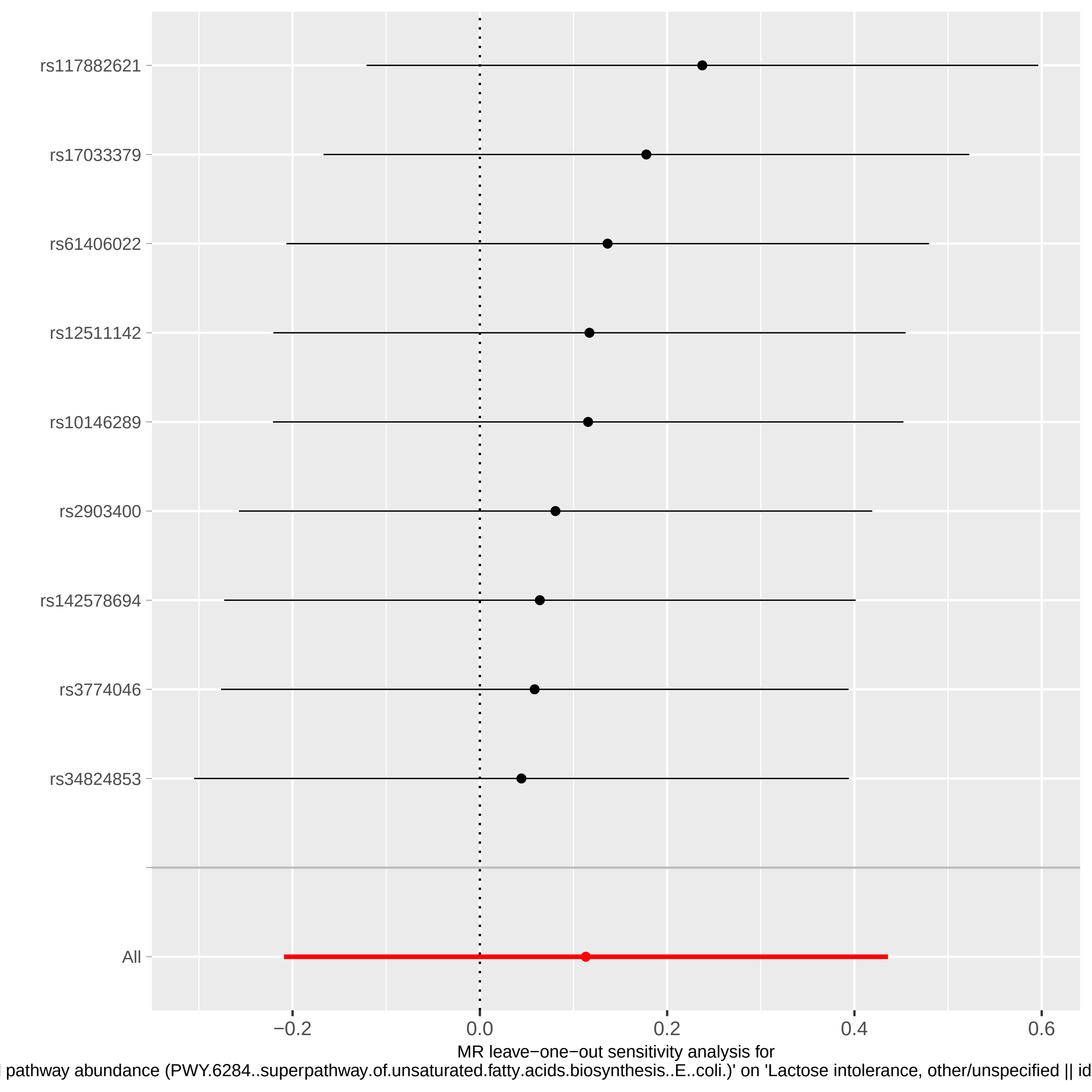
rs117882621
rs17033379
rs61406022
rs12511142
rs10146289
rs2903400
rs142578694
rs3774046
rs34824853
All
−0.2
0.0
0.2
0.4
0.6
MR leave−one−out sensitivity analysis for
l pathway abundance (PWY.6284..superpathway.of.unsaturated.fatty.acids.biosynthesis..E..coli.)' on 'Lactose intolerance, other/unspecified || id
